# Acupuncture for perimenopausal depression: A protocol for a systematic review and meta-analysis

**DOI:** 10.1097/MD.0000000000014073

**Published:** 2019-01-11

**Authors:** Xiao Xiao, Jiayuan Zhang, Yuxia Jin, Yunxia Wang, Qi Zhang

**Affiliations:** Chengdu university of traditional Chinese medicine, Chengdu, Sichuan province, China.

**Keywords:** acupuncture, perimenopausal depression, randomized controlled trial, systematic review

## Abstract

**Background:**

Depression is one of common disease in the female perimenopausal period. It deprives women of their right to work and live normally, and even destroys the happiness of their families. Acupuncture is a promising treatment for perimenopausal depression.

**Methods:**

Cochrane Central Register of Controlled Trials (CENTRAL), PubMed, EMBASE, China National Knowledge Internet (CNKI), Chongqing VIP (CQVIP), Wanfang Data, and on-line trial registries such as ClinicalTrials.gov (ClinicalTrials.gov/), European Medicines Agency (EMA)(www.ema.europa.eu/ema/), WHO International Clinical Trials Registry Platform (www.who.int/ictrp) will be searched from establishment of the database until Oct. 2018. There are no restrictions on the language of publication. The randomized controlled trials of acupuncture (electroacupuncture and manual acupuncture) for perimenopausal depression will be included, and all articles will be screened and collected by 2 reviewers independently. Revman 5.3.5 software will be used for meta-analysis. The specific process will refer to the Cochrane Handbook for Systematic Review.

**Results:**

The efficacy and safety of acupuncture for perimenopausal depression will be comprehensively assessed from the outcomes, including the effective rate, HAMD score, estrogen level and incidence of adverse events.

**Conclusion:**

This systematic review will provide evidence for whether acupuncture can improve perimenopausal depression.

**Ethics and dissemination:**

There is no requirement of ethical approval, and the review will be reported in a peer-reviewed journal.

## 1 Introduction

Depression is one of the most common mental diseases in modern society. According to a global statistic, the prevalence of depression had reached 3.2%^[1]^. A survey showed that from 2005 to 2015, the prevalence of depression in the United States increased significantly^[2]^. Meanwhile, a study in Leipzig, Germany, showed that the prevalence of depression symptoms in adults was 6.4%^[3]^, and the prevalence in women was significantly higher than that in men^[3–5]^. Perimenopausal women have a higher risk of depression, and the severity of symptoms is higher than that any other period ^[6]^. A survey conducted in Shanghai, China, showed that the incidence rate of depression among perimenopausal women is as high as 25.99%^[7]^.

Depression symptoms are main clinical manifestations of perimenopausal depression. The specific manifestations are that the patient's happiness index declined, depression, despair, even suicidal tendency, and often accompanied by dysfunction symptoms of endocrine and autonomic nervous. However, there is no clear understanding of the etiology of perimenopausal depression,^[8]^ which is generally believed to be related to marriage, family, economic status and education level. According to the high incidence of perimenopausal depression and the characteristics of hormone fluctuations during this period, more and more scholars believe that the cause for the high incidence of depression in perimenopausal period may be related to the large fluctuations of hormones. But how hormone fluctuations contribute to the heightened risk is not fully understood, researchers speculate that it may involve intrinsic functional connectivity.^[9,10]^ Therefore, in addition to psychotherapy and antidepressants, hormone replacement therapy (HRT) and antidepressant combined with HRT are also used for the treatment of perimenopausal depression. However, it is controversial that HRT for perimenopausal depression.^[11]^ HRT of any form is an ineffective antidepressant in women who are well into the postmenopausal period.^[12,13]^ Moreover, the efficacy of antidepressant drugs also cannot meet the patient's expectations, and side effects such as dry mouth, fatigue, drowsiness, weight gain, and sexual dysfunction, often lead to poor patient compliance.^[14,15]^

In China, acupuncture is one of the most common therapeutic methods for perimenopausal depression. As well as the clinical reports on acupuncture for perimenopausal depression have gradually increased in recent years. However, there is a lack of systematic review and meta-analysis. The research will objectively assess the evidence from clinical randomized controlled trials (RCTs) on acupuncture for perimenopausal depression.

## 2 Methods

### 2.1 Registration

This systematic review protocol has been registered on PROSPERO as CRD42018114506. In this paper, the protocol will be performed using the methods introduced in the *Cochrane Handbook for Systematic Reviews of Intervention*^[16]^ and reported according to the PRISMA-P guidelines.^[17]^ If we will refine procedures described in this protocol, we will document the amendments in the PROSPERO database and disclose them in future publications related to this meta-analysis.

### 2.2 Eligibility criteria for considering studies

#### 2.2.1 Types of studies

All randomized controlled trials (RCTs) on acupuncture for perimenopausal depression will be included, regardless of whether the blind method is used or not. Language and publication time are unlimited. Small sample size (n < 10) and repeatedly published articles should be excluded.

#### 2.2.2 Types of participants

Perimenopausal women diagnosed with depression are included. The time range of perimenopausal period is based on the 2012 criteria of the North American Menopause Society.^[18]^ And the diagnosis of depression should meet one of the criteria of ICD-10,^[19]^ DSM-5^TM^^[20]^ and CCMD-3.^[21]^ Patients with other serious diseases such as heart disease, stroke, and severe skin problems will be excluded. Baseline is uniform for all participants in each RCT.

#### 2.2.3 Types of interventions

The experimental group should be treated with manual acupuncture or electroacupuncture, and acupuncture points are not restricted. But acupuncture combined with drug therapy will be excluded. The control group should adopt one of the following treatment methods: antidepressant, antidepressant plus HRT, placebo, sham acupuncture. It is acceptable that the treatment last at least 2 weeks. In addition to intervention measures, other treatment and nursing measures should be consistent between the 2 groups.

#### 2.2.4 Types of outcome measures

##### 2.2.4.1 Primary outcome

(1) Effective rate. The criteria for effective treatment are based on the HAMD score reduction rate of more than 25%.^[22]^ (2) HAMD score. The lower the HAMD score was, the more significant effect the interventions had.

##### 2.2.4.2 Secondary outcome

Estrogenic hormone level (FSH, E2, LH), HAMD score during follow-up period, incidence of adverse events.

### 2.3 Search methods for identifying the studies

#### 2.3.1 Data sources and searches

Cochrane Central Register of Controlled Trials (CENTRAL), PubMed, EMBASE, China National Knowledge Internet (CNKI), Chongqing VIP (CQVIP), Wanfang Data, and on-line trial registries such as ClinicalTrials.gov (ClinicalTrials.gov/), European Medicines Agency (EMA) (www.ema.europa.eu/ema/), WHO International Clinical Trials Registry Platform (www.who.int/ictrp) will be searched from establishment of the database until Oct. 2018. There is no restriction on the language of publication

The key search terms used are [(“perimenopaus∗” OR “climacter∗” OR “menopaus∗” OR “premenopaus∗” OR “postmenopaus∗”) AND (“depression” OR “depressive disorder∗” OR “melancholia”)] AND (“acupuncture” OR “electroacupuncture” OR “needl∗” OR “acupoint∗” OR “acupuncture point∗”) AND (“randomized controlled trial” OR “randomized clinical trial”).

### 2.4 Study selection and data extraction

**Figure 1:**
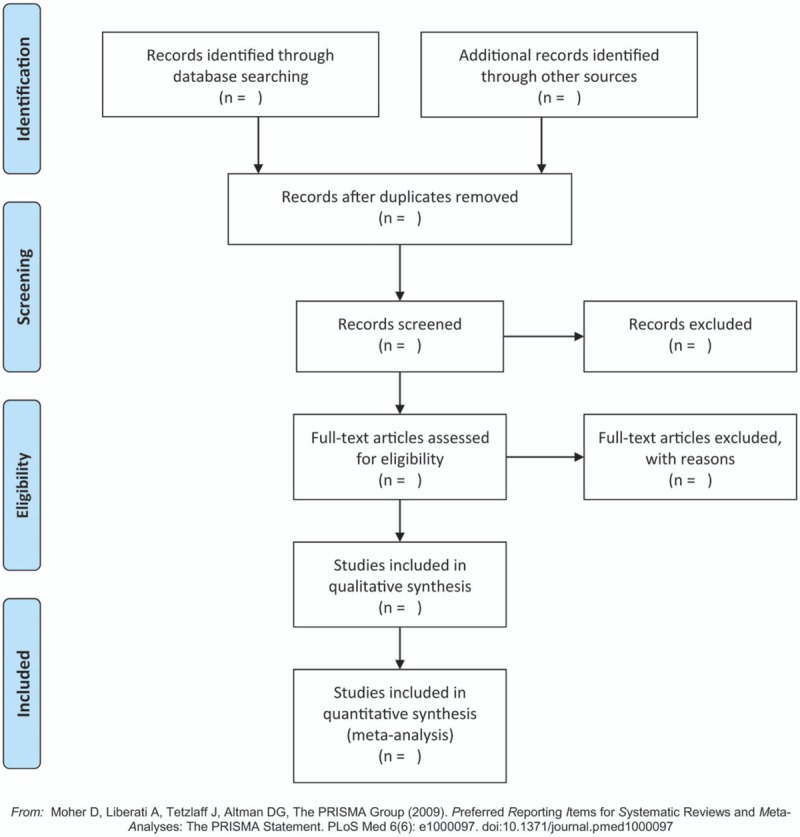
Flow diagram of the study selection process.

Two reviewers (Xiao Xiao and Jiayuan Zhang) independently screen and collect articles. Whether an article will be included or excluded according to the above criteria. The reasons for excluding any article should be noted. If there are different opinions between 2 reviewers, it shall be resolved by discussion or seeking help from a third reviewer (Yuxia Jin). If the consensus still cannot be reached, the dispute shall be settled by contacting the original author for original data. The specific process of study selection is shown in Fig. 1.

After the articles included are ultimately determined, a predefined data template will be prepared, which includes following information: characteristics of the study, participants, intervention, outcome measures, adverse events, and follow-up date. The template is presented to 2 reviewers (Xiao Xiao and Jiayuan Zhang), who will independently extract and code the data based on the template respectively. Finally, the data obtained by the 2 reviewers will be checked each other. If there is a dispute, discussion will be the best way to solve it. If the data are missing, it can be obtained by contacting the original author or transformation of existing data.

### 2.5 Assessment of risk of bias

Two researchers (Xiao Xiao and Yunxia Wang) assess the risk of bias independently, using a collaboration tool recommended by the Cochrane Handbook 5.1.^[16]^ There are 6 points that should be evaluated: random allocation, allocation concealment, blinding, incomplete outcome data, selective outcome reporting and other biases. Disagreement will also be settled by discussion.

### 2.6 Data analysis

#### 2.6.1 Date synthesis

RevMan 5.3.5 software provided by Cochrane collaboration (www.cochrane.org) will be used to conduct meta-analysis and synthesis. Risk ratio (OR) and 95% confidence interval (95% CI) will be used for dichotomous variable; mean difference (MD) and 95% confidence interval (95% CI) will be used for continuous variable; standardized mean difference (SMD) and 95% confidence interval (95% CI) will be used for continuous variable when the units are different. It is considered statistically significant when *P* < .01.

#### 2.6.2 Assessment of heterogeneity

In order to assess heterogeneity, we use the chi-square test and I^2^ statistic. When I^2^ > 50% or *P* < .10, it indicates that the heterogeneity exceeds the acceptable range. If the heterogeneity is small, in the acceptable range (*P* > .10, I^2^ < 50%), the fixed effect model is used for data analysis; otherwise, the random effect model is used.

#### 2.6.3 Subgroup analysis and sensitivity analysis

Subgroup analysis and sensitivity analysis will also be employed to explore the possible causes of heterogeneity. Subgroup analysis will be based on possible factors that may lead to heterogeneity, such as intervention measures (electroacupuncture and manual acupuncture), control measures, length of treatment or quality of articles, etc. If quantitative synthesis is not appropriate, we will conduct a narrative synthesis.

### 2.7 Assessment of publication bias

If more than 10 articles are included, publication bias will be analyzed by visual inspection of funnel plots. A symmetrical distribution of funnel plot data indicates that there is no publication bias.

### 2.8 Confidence in cumulative evidence

GRADE system will be used for assessing the strength of the body of evidence.^[23]^ According to the grading system, the quality of evidence will be rated high, moderate, low and very low.

## 3 Discussion

Depression is one of common diseases in the perimenopausal female. For its treatment, psychological counseling and antidepressant drug are the main methods. Acupuncture is a common treatment for depression in China, and according to a survey conducted in the United States, depression ranks second among the top 10 indications of acupuncture.^[24]^ Therefore, acupuncture is a promising treatment for depression. Acupuncture has the advantages of simple, convenient, cheap and fewer side effects, so it is easily accepted by patients. But is acupuncture effective for hormone-fluctuating perimenopausal depression? This study will conduct a meta-analysis of related RCTs, and provide the current evidence on the efficacy and safety of acupuncture for perimenopausal depression, so as to better guide clinical practice.

## Author contributions

Xiao Xiao, Qi Zhang designed the systematic review. Qi Zhang is the guarantor of the article. The protocol was drafted by Xiao Xiao. Qi Zhang revised the manuscript. Xiao Xiao and Jiayuan Zhang will screen the titles, abstracts, keywords of all retrieved records and extract data independently. XiaoXiao and Yunxia Wang will assess the ROB independently. Yuxia Jin arbitrated in cases of disagreement and ensured the absence of errors. All review authors approved the publication of the protocol.

**Conceptualization:** Xiao Xiao.

**Data curation:** Jiayuan Zhang, Yunxia Wang.

**Formal analysis:** Xiao Xiao, Jiayuan Zhang, Yuxia Jin.

**Investigation:** Xiao Xiao.

**Methodology:** Xiao Xiao.

**Project administration:**Xiao Xiao, Qi Zhang.

**Software:** Xiao Xiao, Yunxia Wang.

**Supervision:** Qi Zhang.

**Writing – original draft:** Xiao Xiao, Yuxia Jin.

**Writing – review & editing:** Xiao Xiao.
